# Melatonin for delirium prevention in acute medically ill, and perioperative geriatric patients

**DOI:** 10.1002/agm2.12112

**Published:** 2020-05-17

**Authors:** Demi R. Asleson, Ada W. Chiu

**Affiliations:** ^1^ Faculty of Pharmaceutical Sciences The University of British Columbia Vancouver British Columbia Canada; ^2^ Surrey Memorial Hospital Fraser Health Authority Surrey British Columbia Canada

**Keywords:** delirium, geriatrics, melatonin

## Abstract

Delirium is a challenging neuropsychiatric ailment that has a negative impact on morbidity and mortality and is difficult to treat once it has developed. The purpose of this review was to analyze the efficacy of melatonin in the prevention of delirium in hospitalized geriatric patients in the acute medically ill and perioperative wards. The databases searched included PubMed (1946 to February 12, 2020), CINAHL (1982 to February 12, 2020), EMBASE (1974 to February 12, 2020), and Web of Science (1900 to February 12, 2020) using search terms related to melatonin, delirium, and prevention. Meta‐analyses, randomized controlled trials, and observational studies were included. We excluded publications pertaining to the intensive care unit or oncology, case reports/series, and those not in English. Seven full‐text publications were included for qualitative analysis. Patient comorbidities, patient medications, melatonin dosing, dosing regimens, and duration of treatment varied between the studies, which yielded heterogeneous results. Overall, this literature review yielded four studies that showed positive results and three that showed negative results for delirium prevention. The current data for the use of melatonin in delirium is conflicting. This area requires further research of more homogeneous studies with larger sample sizes.

## 1 BACKGROUND

Delirium is a syndrome of neuropsychiatric signs and symptoms that can go hand in hand with many serious medical conditions seen and treated in the hospital.^1^ The *Diagnostic and Statistical Manual of Mental Disorders*, 5th edition (DSM‐5) defines “delirium” as a disturbance of consciousness and a change in cognition that develops over a short period of time with fluctuations of the status.^2^ Up to 25% of hospitalized patients may develop delirium and this risk increases to greater than 50% in the geriatric population.^3^ Delirium has been found to be associated with poor outcomes in the elderly regardless of age, comorbid illness, sex, or baseline dementia.^4^ Many studies have found delirium to be linked to impaired cognitive and physical function. Delirium has also been shown to impair functional recovery postoperatively^5^, ^6^ and there is evidence that delirium is associated with increased risk of mortality.^7^ In the United States, over US $164 billion in annual health‐care cost was attributed to delirium in the elderly.^8^, ^9^ Hence, delirium prevention has been of immense research interest in recent years. Many agents have been studied for their effectiveness in the prevention of delirium, including benzodiazepines, antipsychotics, acetylcholinesterase inhibitors, alpha 2‐agonists, melatonin, and melatonin receptor agonists.^10^ However, there has been little success in finding an effective agent for the prevention of delirium in the hospital setting.

Hospitalized patients often have disruption in their circadian rhythm upon admission to hospital. The hospital environment is always active with noise and light and can be stressful or unsettling for a patient, which can be detrimental to a patient's sleeping cycle. Disruption of sleep and circadian rhythm has been found to be a risk factor for the development of delirium.^10^ Melatonin is an endogenous hormone synthesized from tryptophan, and is an important regulator of the circadian rhythm. Melatonin is normally released later in the evening when stressors are low and there is less sunlight exposure.^10^ It is postulated that supplementation of melatonin in the hospital setting to help mimic the circadian rhythm may aid in preventing delirium in patients at risk.^11^ In addition, there have been observations that elderly patients have disturbed melatonin secretion patterns, and that patients with high risk of delirium may have lower levels of melatonin.^12^, ^13^ All of these factors point to the potential role of melatonin in delirium. The purpose of this review was to summarize the current evidence for melatonin on its efficacy, use, and dosing for preventing delirium in hospitalized elderly patients.

## 2 LITERATURE SEARCH

### 2.1 Search strategy

A literature search was performed using PubMed (1946 to February 12, 2020), CINAHL (1982 to February 12, 2020), EMBASE (1974 to February 12, 2020), and Web of Science (1900 to February 12, 2020). The search terms used were “melatonin,” “delirium,” and “prevention.” The search was limited to English‐language publications only. Articles yielded from the search were then analyzed against this review's inclusion criteria by their titles, abstracts, and full texts. A manual review of the references of the available literature was performed with relevant literature included.

### 2.2 Study selection and data extraction

**Figure 1:**
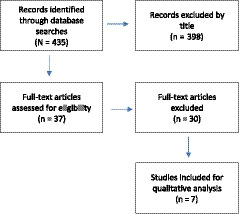
PRISMA flow diagram of study selection following the specific inclusion and exclusion criteria for this review

For the purpose of this review, only original articles on geriatric patients in the acute medically ill and perioperative patient populations were included. The geriatric population included any individual who was ≥ 65 years old. The acute medically ill included any patient with a medical condition that had presented with a severe and abrupt onset that was expected to resolve within approximately 6 months. Lastly, “perioperative” was defined as the time period of a patient's surgical procedure, including admission, anesthesia, surgery, and recovery. Meta‐analyses, randomized controlled trials (RCTs), and observational studies were included in this review. Studies conducted in the intensive care unit and oncology units were excluded. The study inclusion process is outlined in the PRISMA flow diagram in Figure 1.

## 3 RESULTS

A total of one meta‐analysis,^11^ five RCTs,^14^, ^15^, ^16^, ^17^, ^18^ and one observational study^19^ were included in this analysis. A summary of the studies is presented in Table 1.

**Table 1:** Studies of melatonin in acute medically ill and perioperative geriatric patients in delirium prevention

Reference	Year	Study design	Intervention dosage/sample size	Comparator/sample size	Timing	Setting	Age (years)	Primary outcome	Assessment tool	Outcome
Jaiswal et al^15^	2018	RCT, blinded, ITT analysis	Melatonin 3 mg/n = 43	Placebo/n = 44	Nightly for 14 nights	Inpatients, internal medicine ward	81.2 ± 7.3 (melatonin), 80.1 ± 8.3	Delirium occurrence	CAM	Melatonin 22.2% (8/36) vs placebo 9.1% (3/33) *P* = 0.19, NSS
Al‐Aama et al^16^	2011	Placebo‐controlled trial, double blind	Melatonin 0.5 mg/n = 61	Placebo/n = 61	Prior to sleep between 6 pm and midnight	Inpatients, emergency department to the internal medicine ward	84.3 ± 5.9 (melatonin), 84.6 ± 6.2 (placebo)	Delirium incidence	CAM and MDAS	Melatonin 12%, placebo 31% *P* = 0.01
Artemiou et al^19^	2015	Prospective clinical observational study	Melatonin 5 mg/n = 250	Placebo/n = 250	Night before cardiac surgery	Inpatients, cardiac surgery	65.2 ± 10.3 (melatonin), 64.3 ± 10.1 (control)	Incidence of postoperative delirium	CAM‐ICU	Placebo 20.8% vs melatonin 8.4% *P* = 0.001
Sultan et al^17^	2010	RCT, double blinded	Melatonin 5 mg/n = 53	Placebo/n = 49, midazolam 7.5 mg/n = 50 or clonidine 100 µg/n = 51	Night before operation	Inpatient, scheduled hip arthroplasty spinal anesthesia	Control: 72.3 ± 6.4 Melatonin: 70.4 Midazolam: ±8.2 Clonidine: ±6.8	Delirium rate	AMT	Placebo 32.65% vs melatonin 9.43% *P* = 0.003 Midazolam and clonidine groups, NSS
De Jonghe et al^14^	2014	RCT, multicenter, double blinded	Melatonin 3 mg/n = 186	Placebo/n = 192	Nightly around 9 pm for 5 days	Inpatients with emergency hip fracture	84.1 ± 8 (melatonin), 83.4 ± 7.5	Incidence of delirium	DSM‐5	Melatonin 29.6% vs placebo 25.5% *P* = 0.4
Chen et al^11^	2016	Meta‐analysis Four RCTs included	Melatonin 5 mg, 0.5 mg, 3 mg	Placebo/NA	Nightly, daily between 6 pm and midnight	Hospital admission to a medical ward	NA	Incidence of delirium	AMT, CAM, DSM‐5	Melatonin groups vs placebo RR 0.41; 95% CI, 0.15‐1.13, NSS
Ford et al^18^	2020	RCT, double blind, placebo‐controlled, ITT analysis	Melatonin 3 mg/n = 105	Placebo/n = 105	At night, started 2 days before surgery, continued 7 days	Inpatient, major cardiac surgery	69 (melatonin group) and 67.6 (placebo), SD 8	Incidence of delirium	CAM	Melatonin 21.4% (21/98) vs placebo 20.2% (21/104) OR = 0.78; 95% CI, 0.35‐1.75, NSS

Abbreviations: AMT, Abbreviated Mental Test; CAM, Confusion Assessment Model; CI, confidence interval; DSM‐5, *Diagnostic and Statistical Manual of Mental Disorders*, 5th edition; ICU, intensive care unit; ITT, intention‐to‐treat; MDAS, Memorial Delirium Assessment Scale; NSS, not statistically significant; OR, odds ratio; RCT, randomized controlled trial; RR, relative risk; SD, standard deviation.

The studies were grouped into either acute medically ill or perioperative, given the heterogeneity of these two populations. The number of patients in each study varied from 161 patients to 500 patients, who were split into intervention and comparator groups. All of the studies compared melatonin to placebo with the exception of Sultan et al,^17^ which compared melatonin to midazolam 7.5 mg, clonidine 100 µg, and a placebo group. Delirium was assessed with a number of different assessment tools, with the most common being the Confusion Assessment Model (CAM).^11^, ^15^, ^16^, ^18^ There were varying results reporting the efficacy of melatonin in the prevention and treatment of delirium. Three studies^14^, ^15^, ^18^ had negative results, suggesting melatonin is not associated with the prevention of delirium in hospitalized and postoperative patients. In contrast, four studies^11^, ^16^, ^17^, ^19^ yielded positive results, showing a decrease in delirium rates in melatonin intervention groups.

### 3.1 Dosing of melatonin

Doses of melatonin used in studies ranged between 0.5 mg and 5 mg once daily. There was variability of when the melatonin was administered: For patients undergoing hip arthroplasty, melatonin was administered the night before the operation, again at 90 minutes before the surgery, and then for 5 consecutive days postoperatively.^14^ In contrast, for patients admitted to the internal medicine ward in Al‐Aama et al,^16^ melatonin was administered prior to sleep between 6:00 pm and midnight for 14 days.

### 3.2 Acute medically ill geriatric patients

There was conflicting evidence on the association between melatonin administration and delirium prevention in the acute medically ill geriatric population. In a study on geriatric patients admitted to an internal medicine ward, Jaiswal et al^15^ found that differences in delirium rates in the melatonin group versus placebo were statistically insignificant (22.2% vs 9.1%, *P* = 0.19). In contrast, Al‐Aama et al^16^ found positive results for melatonin decreasing delirium rates in emergency patients admitted to the internal medicine ward using the CAM (31% vs 12%, *P* = 0.01). The Memorial Delirium Assessment Scale (MDAS) was used to assess delirium severity, and there was no statistically significant difference between groups.^16^ Chen et al^11^ carried out a meta‐analysis of four RCTs, three of which are included in this review (de Jonghe et al,^14^ Al‐Aama et al,^16^ and Sultan et al^17^) and one of which met our exclusion criteria due to its analysis of intensive care unit patients and assessment of ramelteon’s efficacy.^20^ Chen et al^11^ analyzed Al‐Aama et al^16^ and Sultan et al^17^ in combination and the results showed a significant reduction in the incidence of delirium (relative risk [RR], 0.30; 95% confidence interval [CI], 0.17‐0.53; *P* < 0.0001). They reported moderate quality of evidence in these analyses, with a “serious” risk of bias.

### 3.3 Perioperative geriatric patients

De Jonghe et al^14^ studied geriatric patients undergoing hip fracture surgery and found no statistically significant difference between melatonin and the placebo group in regards to delirium rates using the DSM‐5 criteria^2^ (29.6% vs 25.5%, *P* = 0.4). Artemiou et al analyzed post‐cardiac surgery patients and found that the difference in delirium rates in the two study groups was statistically significant (placebo 20.8% vs melatonin 8.4%, *P* = 0.001).^19^ Sultan et al (n = 222, median age ≥ 65 years) looked at melatonin treatment preoperatively in hip arthroplasty patients; their results revealed a statistically significant decrease in rates of delirium in the melatonin intervention groups (placebo 32.65% vs melatonin 9.43%, *P* = 0.003).^17^ Ford et al^18^ analyzed patients undergoing major cardiac surgery and found no statistically significant difference between groups (melatonin 21.4% vs placebo 20.2%; 95% CI, 0.35‐1.75). Additionally, Chen et al^11^ analyzed Al‐Aama et al,^16^ de Jonghe et al,^14^ and Sultan et al,^17^ which included a perioperative population in the study by de Jonghe et al. They found a statistically insignificant decrease in delirium incidence with melatonin supplementation (RR, 0.53; 95% CI, 0.20‐1.43; *P* = 0.21).^11^

## 4 DISCUSSION

The geriatric population is at higher risk of developing delirium as well as other complications, including fall risk, frailty, and sensitivity to medications, given their pharmacokinetics. In the studies reviewed, the DSM‐5 criteria for delirium, the CAM, the MDAS, and the Abbreviated Mental Test were used solely or in different combinations to assess presence of delirium in patients. The DSM‐5 is considered to be the gold standard for assessment and diagnosis of delirium in medical patients; however, it requires a trained psychiatric professional and is too cumbersome for most situations.^21^ The CAM, which can be completed in 5 minutes by non‐psychiatric trained professionals, is the most widely accepted alternative to the DSM‐5 in assessing delirium, given its ease of use at the bedside and has been well validated in multiple studies. The Abbreviated Mental Test is considered a part of the delirium assessment but not a complete assessment of the condition.^22^ The MDAS was used to assess delirium severity only. Each of the tools for delirium diagnosis analyses different characteristics of the patient, including inattention and consciousness; cognitive and emotional dysfunction; perceptional dysfunction and delusions; psychomotor features; and the patient's overall function.^21^ The lack of standardization of assessment tools used creates a barrier for comparing the results of the studies in terms of delirium diagnosis and also gives rise to doubts about the validation of the results.

In the studies conducted in acute medically ill geriatric populations, there were conflicting results. Jaiswal et al found a non‐statistically significant trend towards lower delirium rates in placebo groups.^15^ This indicates that melatonin could be an ineffective agent or that the study lacked power due to small sample size and/or heterogeneity in their treatment groups. Patients were assessed for delirium twice daily by the floor nurses, which could have introduced interrater variability. In contrast, Al‐Aama et al^16^ found melatonin to have lower delirium rates than the placebo group. Patients were assessed with the CAM every 24‐48 hours, which introduced variability in the number of data points per patient and could have yielded skewed results. Also, one of the features of delirium is fluctuation of mental status, which could have been missed if the CAM assessment had been conducted during a prolonged assessment interval.

The studies in the perioperative geriatric population were equally as conflicting. The results found by de Jonghe et al did not show a statistically significant difference in delirium rates between the melatonin and the placebo group and they concluded that melatonin was not associated with the prevention of delirium.^14^ In addition, de Jonghe et al showed that there was no difference between the groups in terms of mortality or in cognitive functional outcomes at the 3‐month follow‐up. It is also important to note that if the participants in the study were taking psychiatric medications prior to entering the study, they could continue their prescription during the study. In addition, 55.6% of the patient population studied had cognitive impairment before admission. This could have predisposed the patients to developing delirium. The study by Ford et al^18^ showed similar results. Ford et al included patients who were 50 years and older; this study was still included in this review because the median ages of the participants were 69 years (melatonin group) and 67.6 years (placebo) with a standard deviation of 8 years. Most of the clinical comorbidities seen in the melatonin and placebo groups were evenly distributed across the groups. In contrast, the studies by Artemiou et al^19^ and Sultan et al^17^ showed a statistically significant decrease in the incidence of delirium with melatonin. In the study conducted by Artemiou et al, it is important to note that there was a higher proportion of psychiatric disease in the placebo group. This could have increased the risk of delirium development and other comorbidities given the imbalance of medical conditions between the groups. Sultan et al^17^ presented a different assessment of delirium prevention, with four study groups in their analysis. The purpose of the clonidine and midazolam groups was to assess delirium development in the presence of other psychotropic medications. They showed positive delirium prevention in the melatonin intervention groups compared to placebo, but no difference in delirium incidence rates. Of note, there is significant heterogeneity between the populations of the three studies, as one involved emergency hip fracture surgery, one involved scheduled hip arthroplasty, and the third involved cardiac surgery,^17^, ^18^, ^19^ which makes it difficult to congregate the findings.

Chen et al presented a meta‐analysis that showed melatonin decreases the incidence of delirium.^11^ When Chen et al combined three of the articles presented in this review, analyzed together the results were statistically insignificant. However, Chen et al^11^ separated out de Jonghe et al^14^ and analyzed Al‐Aama et al^16^ and Sultan et al^17^ together. These results showed that melatonin decreased delirium incidence by 70% relatively. This shows a stronger association than the individual articles. In addition, it was reported that there was “serious” bias in these results as well as heterogeneity (*I* ^2 ^= 84.0%, *P* = 0.0003).^11^

Overall, these data suggest that there may be an association between melatonin and prevention of delirium. The contradictions in the data may be due to the lack of power in the studies due to small sample sizes. Other reasons may be heterogeneity in the patient populations studied, including chronic medications, indication for being admitted, and comorbidities. The studies were separated into the two groups given the difference in etiology of delirium development. Infection and sepsis likely present as major contributing factors to delirium in acute medically ill patients, whereas this would be unlikely for surgical patients. Most of the studies presented were double blinded, and used intention‐to‐treat analysis and a standardized delirium assessment tool. Overall, the studies presented had relatively small sample sizes and did not always have statistically significant results, possibly due to a lack of power to detect the effect of melatonin in these populations. The lack of standardization in assessment also adds to the difficulty in interpreting the results. Also, given the small sample sizes and variability of patient comorbidities, the results are only partially generalizable to the patient populations of focus. In addition to comorbidities, other drug therapies that the patients were taking before they were admitted to the hospital were not always reported. This created potential for confounders that could have affected the results seen.

In the face of these results, the efficacy of melatonin for delirium prevention is not yet clear. The use of standardized assessment tools (such as the DSM‐5 and the CAM), larger multicenter RCTs, and standardized melatonin dosing is strongly recommended to properly detect the effect of melatonin on delirium incidence. In current practice, melatonin is not routinely used as a delirium prevention agent, but it is used for other purposes, such as a sleep aid. This may enable a larger study sample to be assessed with one of the standardized diagnostic tools. In addition, an effective dose should be established, as well as a dosing interval and a time interval for evaluating delirium with a diagnostic tool.

### 4.1 Ramelteon

Ramelteon is a melatonin receptor agonist that has similar action to exogenous melatonin. Ramelteon is found to have a high affinity and selectivity for melatonin receptors 1 and 2 versus melatonin.^23^ Ramelteon has been approved by the US Food and Drug Administration for the treatment of insomnia and has been studied as an option for preventing delirium in a case series.^24^ In a randomized placebo‐controlled trial, ramelteon was associated with a statistically significant decrease in incidence of delirium from 32% to 3% (*P* = 0.003) in acute care. There are some meaningful studies showing evidence for this agent, and this would help get closer to finding an agent that can help with the prevention of delirium in acute medically ill, geriatric, and perioperative patients. While our review was focused on melatonin specifically, ramelteon is an agent of similar interest. This agent is not currently available in Canada or Europe.

### 4.2 Why should we continue to study melatonin?

Delirium can have mortality and morbidity consequences in the geriatric population admitted to hospital, given their comorbidities and frailty. These patients are likely to be subjected to polypharmacy and are at high risk of medications’ adverse effects and drug‐drug interactions.^25^, ^26^ Many of the medications studied in the prevention of delirium have not been found to be effective, and oftentimes they are on the American Geriatrics Society Beers Criteria list as having a high potential of complications in geriatrics.^27^ Also, once delirium develops, geriatric patients are often given medications such as benzodiazepines and antipsychotics, which have been associated with worse delirium and increased morbidity and mortality.^28^, ^29^, ^30^ Melatonin, with its relatively high safety and tolerability profile, has been used widely for sleep disorders. While its role for delirium prophylaxis remains unclear, it remains an attractive research subject for delirium, especially in the geriatric population.

## 5 CONCLUSION

Overall, our results suggest that melatonin possibly plays a role in delirium prevention in hospitalized and postoperative geriatric patients; however, the results are conflicting. Inconsistencies in methodologies, assessment tools, and dosing result in much heterogeneity in the evidence. It may be reasonable to direct research efforts in a more focused subset of patients who are at higher risk of developing delirium. Regardless, in order to determine the efficacy of melatonin in this population, more RCTs with larger sample sizes and more sophisticated study designs are needed.

## CONFLICTS OF INTEREST

We have no conflicts of interest for conducting this review.

## AUTHOR CONTRIBUTIONS

Both authors: writing of the paper, substantially contributed to conception or design, gave final approval. Demi R. Asleson: drafted the manuscript, contributed to acquisition and interpretation of data. Ada W. Chiu: critically revised the manuscript for important intellectual content.

## References

[1] Schwartz AC , Fisher TJ , Greenspan HN , Heinrich TW . Pharmacologic and nonpharmacologic approaches to the prevention and management of delirium. Int J Psychiatry Med. 2016;51(2):160‐170. 26941206 10.1177/0091217416636578

[2] American Psychiatric Association . *Diagnostic and Statistical Manual of Mental Disorders*. 5th ed. Washington, DC: American Psychiatric Association; 2013.

[3] Vasilevskis EE , Han JH , Hughes CG , Ely EW . Epidemiology and risk factors for delirium across hospital settings. Best Pract Res Clin Anaesthesiol. 2012;26(3):277‐287. 23040281 10.1016/j.bpa.2012.07.003 PMC3580997

[4] Witlox J , Eurelings LSM , De Jonghe JFM , Kalisvaart KJ , Eikelenboom P , Van Gool WA . Delirium in elderly patients and the risk of postdischarge mortality, institutionalization, and dementia: a meta‐analysis. J Am Med Assoc. 2010;304(4):443‐451. 10.1001/jama.2010.1013 20664045

[5] Hshieh TT , Saczynski J , Gou RY , et al. Trajectory of functional recovery after postoperative delirium in elective surgery. Ann Surg. 2017;265(4):647‐653. 27501176 10.1097/SLA.0000000000001952 PMC5292310

[6] Saczynski JS , Marcantonio ER , Quach L , et al. Cognitive trajectories after postoperative delirium. N Engl J Med. 2012;367(1):30‐39. 22762316 10.1056/NEJMoa1112923 PMC3433229

[7] Marcantonio ER , Kiely DK , Simon SE , et al. Outcomes of older people admitted to postacute facilities with delirium. J Am Geriatr Soc. 2005;53(6):963‐969. 15935018 10.1111/j.1532-5415.2005.53305.x

[8] Siddiqi N , Harrison JK , Clegg A , et al. Interventions for preventing delirium in hospitalised non‐ICU patients. Cochrane Database Syst Rev. 2016;(3)CD005563. 26967259 10.1002/14651858.CD005563.pub3 PMC10431752

[9] Oh ES , Fong TG , Hshieh TT , Inouye SK . Delirium in older persons: advances in diagnosis and treatment. J Am Med Assoc. 2017;318(12):1161‐1174. 10.1001/jama.2017.12067 PMC5717753 28973626

[10] Ford AH , Almeida OP . Pharmacological interventions for preventing delirium in the elderly. Maturitas. 2015;81(2):287‐292. 25890587 10.1016/j.maturitas.2015.03.024

[11] Chen S , Shi L , Liang F , Xu L , Desislava D , Wu Q . Exogenous melatonin for delirium prevention: a meta‐analysis of randomized controlled trials. Mol Neurobiol. 2016;53(6):4046‐4053. 26189834 10.1007/s12035-015-9350-8

[12] Shigeta H , Yasui A , Nimura Y , et al. Postoperative delirium and melatonin levels in elderly patients. Am J Surg. 2001;182(5):449‐454. 11754849 10.1016/s0002-9610(01)00761-9

[13] Perras B , Kurowski V , Dodt C . Nocturnal melatonin concentration is correlated with illness severity in patients with septic disease. Intensive Care Med. 2006;32(4):624‐625. 16477409 10.1007/s00134-006-0069-x

[14] de Jonghe A , van Munster BC , van Oosten HE , et al. The effects of melatonin versus placebo on delirium in hip fracture patients: study protocol of a randomised, placebo‐controlled, double blind trial. BMC Geriatr. 2011;11:34. 21729284 10.1186/1471-2318-11-34 PMC3148574

[15] Jaiswal SJ , Mccarthy TJ , Wineinger NE , Kang DY , Song J . Melatonin and sleep in preventing hospitalized delirium: a randomized clinical trial. Am J Med. 2018;131(9):1110‐1117. 29729237 10.1016/j.amjmed.2018.04.009 PMC6163056

[16] Al‐Aama T , Brymer C , Gutmanis I , Woolmore‐Goodwin SM , Esbaugh J , Dasgupta M . Melatonin decreases delirium in elderly patients: a randomized, placebo‐controlled trial. Geriatr Psychiatry. 2011;26(7):687‐694. 10.1002/gps.2582 20845391

[17] Sultan S . Assessment of role of perioperative melatonin in prevention and treatment of postoperative delirium after hip arthroplasty under spinal anesthesia in the elderly. Saudi J Anaesth. 2010;4(3):169‐173. 21189854 10.4103/1658-354X.71132 PMC2980663

[18] Ford AH , Flicker L , Kelly R , et al. The Healthy Heart‐Mind trial: randomized controlled trial of melatonin for prevention of delirium. J Am Geriatr Soc. 2020;68(1):112‐119. 31595489 10.1111/jgs.16162

[19] Artemiou P , Bily B , Bilecova‐Rabajdova M , et al. Melatonin treatment in the prevention of postoperative delirium in cardiac surgery patients. Anaesthesiol Intensive Care. 2015;12(2):126‐133. 10.5114/kitp.2015.52853 PMC4550034 26336494

[20] Hatta K , Kishi Y , Wada K , Takeuchi T , Odawara T , Usui C , Nakamura H . Preventive effects of ramelteon on delirium. JAMA Psychiatry. 2014;71(4):397‐403. 10.1001/jamapsychiatry.2013.3320. 24554232

[21] Jones RN , Cizginer S , Pavlech L , et al. Assessment of instruments for measurement of delirium severity: a systematic review. JAMA Intern Med. 2019;179(2):231‐239. 30556827 10.1001/jamainternmed.2018.6975 PMC6382582

[22] Dumanyan E , Skopets AA , Malyshev YP . Does euroscore predict delirium after cardiac surgery? Neurosciences. 2019;31:121.

[23] Neubauer DN . A review of ramelteon in the treatment of sleep disorders. Neuropsychiatr Dis Treat. 2008;4(1):69‐79. 18728808 10.2147/ndt.s483 PMC2515902

[24] Kishi HK , Wada Y , Takeuchi K , Takashi O , Toshinari U , Chie NH . Preventive effects of ramelteon on delirium: a randomized placebo‐controlled trial. JAMA Psychiatry. 2019;71(4):397‐403. 10.1001/jamapsychiatry.2013.3320 24554232

[25] Doucet J , Chassagne P , Trivalle C , Landrin I , Pauty MD , Kadri N . Drug‐drug interactions related to hospital admissions in older adults: a prospective study of 1000 patients. J Am Geriatr Soc. 1996;44(8):944‐948. 8708305 10.1111/j.1532-5415.1996.tb01865.x

[26] Nguyen JK , Fouts MM , Kotabe SE , Lo E . Polypharmacy as a risk factor for adverse drug reactions in geriatric nursing home residents. Am J Geriatr Pharmacother. 2006;4(1):36‐41. 16730619 10.1016/j.amjopharm.2006.03.002

[27] Fick DM , Semla TP , Steinman M , et al. American Geriatrics Society 2019 updated AGS Beers Criteria® for potentially inappropriate medication use in older adults. J Am Geriatr Soc. 2019;67(4):674–694. 30693946 10.1111/jgs.15767

[28] Setoguchi S , Wang PS , Alan Brookhart M , Canning CF , Kaci L , Schneeweiss S . Potential causes of higher mortality in elderly users of conventional and atypical antipsychotic medications. J Am Geriatr Soc. 2008;56(9):1644‐1650. 18691283 10.1111/j.1532-5415.2008.01839.x

[29] Dublin S , Walker RL , Jackson ML , et al. Use of opioids or benzodiazepines and risk of pneumonia in older adults: a population‐based case–control study. J Am Geriatr Soc. 2011;59(10):1899‐1907. 22091503 10.1111/j.1532-5415.2011.03586.x PMC3223721

[30] Madhusoodanan S , Bogunovic OJ . Safety of benzodiazepines in the geriatric population. Expert Opin Drug Saf. 2004;3(5):485‐493. 15335303 10.1517/14740338.3.5.485

